# The effects of series elastic stiffness and cutaneous sensitivity on leg muscle reflex responses to unanticipated slips during walking

**DOI:** 10.1007/s00221-025-07095-8

**Published:** 2025-05-20

**Authors:** Ross E. Smith, Andrew D. Shelton, Gregory S. Sawicki, Jason R. Franz

**Affiliations:** 1 Lampe Joint Department of Biomedical Engineering, UNC Chapel Hill and NC State University, Chapel Hill, USA; 2 George W. Woodruff School of Mechanical Engineering, Georgia Institute of Technology, Atlanta, USA; 3 School of Biological Sciences, Georgia Institute of Technology, Atlanta, USA

**Keywords:** Tendon, Balance, Stability, Geriatrics, Biomechanics, Ankle

## Abstract

Fall-related injuries are a large public health concern for older adults (OA). Delayed or inappropriate afferent signaling may hinder detection of balance perturbation (e.g., slip), predisposing OA to falls and less efficacious corrective actions. Perturbation detection is largely governed by proprioceptive acuity, consisting of sensory information from skin and musculotendinous receptors. While skin and muscle receptors’ signaling thresholds increase with age, feedback from muscle may be uniquely delayed and diminished by age-related decreases in series elastic tissue stiffness which, during a rapid, unanticipated joint position change, could decrease the velocity of muscle stretch, delaying detection. Ultimately, the association between skin and muscle afferent signaling integrity and muscle reflex timing in response to walking balance perturbations has yet to be well established. This study evaluated the associations between plantar cutaneous sensitivity (PS) and Achilles tendon stiffness (k_AT_) to muscle reflex responsiveness of ankle plantar- and dorsiflexor muscles following treadmill-induced slip perturbations in younger and OA. Compared to younger adults (YA), we found that OA had lesser PS and k_AT_ and delayed tibialis anterior excitation onset. However, neither sensory outcome associated with muscle onset times. Later in stance, OA exhibited earlier triceps surae onsets than YA. However, only in YA did earlier gastrocnemius activity associate with greater k_AT_, which may indicate an ankle dominant balance recovery strategy in YA. Our results point to specific age-related changes in the timing of neuromuscular corrections to mitigate instability, which may underlie age-related differences in balance recovery efficacy and subsequent injury severity.

**Supplementary Information:**

The online version contains supplementary material available at 10.1007/s00221-025-07095-8.

## Introduction

Falls among older adults (OA) can result in devastating injuries, decreasing independence and quality of life, and costing as much $50 billion annually in the US alone (Tromp et al. 2001). These costs are expected to increase as the older adult population (65 and older) continues to grow, with as many as one in three OA falling each year (Florence et al. 2018). Research has sought to mitigate these concerns, revealing a number of preventative measures for OA to improve extrinsic (wearing appropriate footwear, avoiding uneven terrain) and intrinsic factors (increasing strength, improving dual-task management) associated with falls (Graafmans et al. 1996; Kelsey et al. 2012; Wollesen et al. 2017). However, in the last decade, fall-related hospitalizations and death rates have increased by 200% and 30%, respectively (CDIS 2022). Thus, the need for novel, modifiable targets to improve OA’ resilience to falls and fall-related injuries after their balance is disrupted is of vital importance to public health.

A major factor influencing OA’ vulnerability to instability and subsequent falls is a decrease in their capacity to execute effective balance recovery strategies. Age-related changes in balance recovery are associated with concomitant decreases in muscle–tendon unit (MTU) mechanical properties (i.e., muscle strength and rate of force development) (Debelle et al. 2022), as well as somatosensory function (i.e., vision (Franz et al. 2015), vestibular integrity (Chiarovano et al. 2017), and proprioceptive acuity (Deshpande et al. 2016)). Immediately following a balance disturbance, somatosensory feedback is required to prompt and inform subsequent muscle excitations and thereby recovery strategies. As balance is frequently disrupted at the foot–ground interface (e.g. 20–50% of falls related to slips and trips (Tinetti et al. 1988; Robinovitch et al. 2013)), compression of cutaneous mechanoreceptors on the foot’s plantar surface of the support limb is likely to precede other somatosensory stimuli, providing rapid afferent signaling and improving reflex responsiveness to perturbed balance. Indeed, prior studies indicate decreased plantar cutaneous sensitivity (PS) in young adults, imposed via intradermal anesthetic solution injections to the plantar foot surface, compromises balance recovery following unanticipated surface translations during standing (Perry et al. 2000). Compared to younger adults (YA), cutaneous sensitivity is diminished in OA as evidenced by greater Semmes–Weinstein monofilament thresholds, which are associated with worse Berg Balance Scale scores and greater postural sway (Peters et al. 2016). Furthermore, OA demonstrate weakened reflexive tibialis anterior (TA) muscle excitation responses (via surface EMG) following mechanical vibratory stimulations to cutaneous receptors on the foot’s plantar surface (Peters et al. 2016). Given that it is well established that OA also demonstrate delayed M- and H-wave reflexes due to age in the gastrocnemius muscles (Sabbahi and Sedgwick 1982; Scaglioni et al. 2003), these findings implicate age-related decreases in plantar sensitivity may further contribute to delayed and/or diminished muscle reflexes and muscle excitation responses from the TA and gastrocnemius muscles, which are vital for regaining balance following gait perturbations (Berger et al. 1984).

Detecting balance perturbation onset is also diminished among OA via age-related reductions in joint, tendon, and muscle proprioceptive acuity. While joint and tendon receptor thresholds are generally met under more extreme joint position or muscle force conditions, more subtle changes in foot and ankle joint positions brought on by a slip or trip result in muscle length changes, which—independent of age—are sensed and relayed to the central nervous system by muscle spindle proprioceptors (Taylor 1972). Muscle spindle integrity is known to decrease with age, worsening joint position sensing accuracy (Adamo et al. 2007). Muscle spindle stretch reflex thresholds are also affected by age, slowing and diminishing muscle excitations to joint position changes (Obata et al. 2012). While age-related reductions in proprioceptive acuity are commonly demonstrated in isolated static postures, OA display similar proprioceptive deficits for the TA, rectus femoris, and rectus abdominus muscles following gait slip perturbations (Tang and Woollacott 1999) and for the soleus muscle following gait trip perturbations (Pijnappels et al. 2005). Diminished stretch reflexes in OA are commonly attributed to decreases in muscle spindle integrity (Liu et al. 2005). However, OA also experience age-related decreases in series elastic tendon stiffness and increases in muscle stiffness (Delabastita et al. 2019; Marcucci and Reggiani 2020). These combined alterations in mechanical properties of a given MTU would, for a given elongation, increase tendon stretch and velocity while reducing muscle—and thereby, muscle spindle—stretch and velocity. The literature concerning this phenomenon is sparse and has mixed results. Blackburn et al. (Blackburn et al. 2008) found no effect on stretch reflex sensitivity between high and low triceps surae MTU stiffness groups among healthy YA. However, increasing muscle and decreasing tendon stiffnesses, particularly for MTUs with compliant tendons, have been implicated in reducing stretch reflex sensitivity during stance in YA (Rack et al. 1983) and following isolated ankle rotations in children and YA (Grosset et al. 2007). Thus, in addition to established degradation of muscle spindle sensitivity with age, muscle reflex responses may be further penalized by age-related decline in MTU structural properties, reducing or slowing muscle spindle afferent discharge and slowing detection of balance perturbations.

Ultimately, while the integrities of plantar cutaneous and muscle spindle receptors are known to degrade with age with concomitant changes in MTU mechanical properties, there is limited literature detailing the functional consequences of these age-related changes on muscle reflex responsiveness following balance perturbation. These rapid reflex responses act as a critical first step in perturbation detection and subsequent recovery responses, which may improve OA ability to prevent or better manage circumstances leading to a fall. Thus, we used this study in a cohort of OA and YA to evaluate the association between (i) plantar cutaneous sensitivity and Achilles tendon stiffness and (ii) muscle excitation responses of the medial gastrocnemius (MG), soleus (SOL), and tibialis anterior (TA) to treadmill-induced slip perturbations. We opted to measure Achilles tendon stiffness (k_AT_) as a surrogate measure for generalized tendon stiffness changes due to age. We first hypothesized that OA would have slower muscle excitation responses for all muscles (MG, SOL, TA) to unanticipated treadmill-induced slip perturbations. We also hypothesized OA would have reduced k_AT_ and greater sensitivity thresholds for Semmes–Weinstein monofilament tests. Finally, we hypothesized that anticipated age-related changes in tendon stiffness and plantar sensation would correlate with delayed muscle excitation responses to slip perturbations. Data in support of these hypotheses would implicate age-related decrements in plantar cutaneous feedback and tendon stiffness in delaying perturbation detection, which may explain greater relative vulnerability to falls and fall-related injuries among OA.

## Methods

### Participants

Twenty-two YA (10 female, age: 21.59 ± 2.10 yrs, height: 1.72 ± 0.09 m, mass: 66.4 ± 8.6 kg) and nineteen OA (11 female, age: 74.05 ± 6.02 yrs, height: 1.68 ± 0.12 m, mass: 68.87 ± 20.18 kg) adults participated. Participants completed the study if they self-reported being comfortable walking 30 min continuously and were excluded if they had a history of neurological disease(s), had a lower limb injury within the last 6 months, or walked with an assistive device. All participants were recruited from the Chapel Hill area or surrounding areas via flyer or word of mouth. All experimental procedures and recruitment procedures were approved by the University of North Carolina at Chapel Hill Institutional Review Board (20–0555), and all participants gave written informed consent prior to participation in the study.

### Experimental procedures

**Fig. 1:**
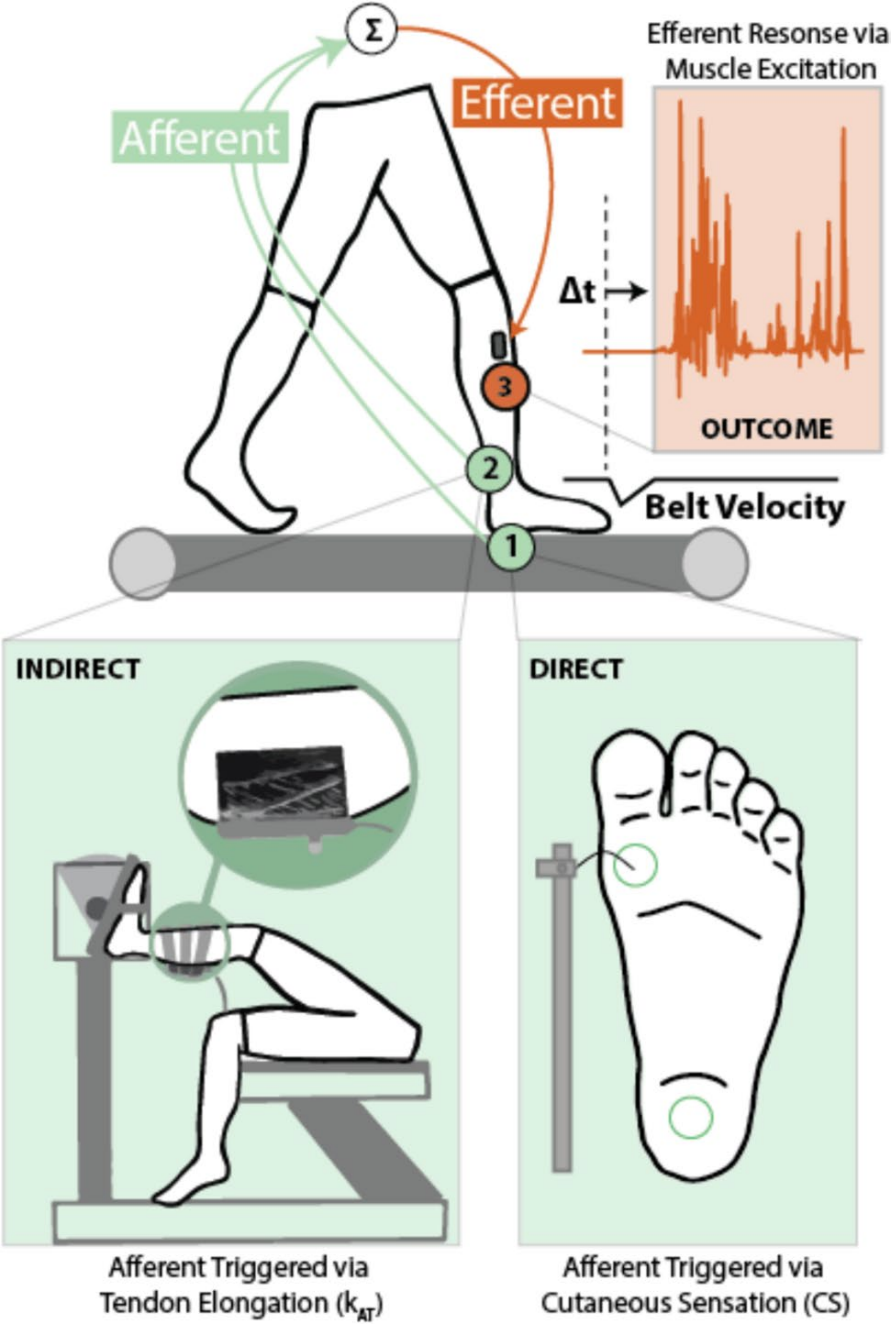
Experimental framework for assessing muscle responses to treadmill-induced slip perturbations. Treadmill belt decelerations are designed to emulate the instability consistent with a slip in the community, resulting first in kinematic changes at the foot and ankle. Mitigating the instability elicited by these perturbations requires afferent detection via: **(1)** plantar cutaneous receptors (e.g., Semmes–Weinstein monofilament thresholds) and **(2)** muscle spindle receptors, which respond to the magnitude and rate of fiber length change and thus the stiffness of series elastic tissues, leading to **(3)** corrective efferent action (muscle excitation response)

Our experimental framework is illustrated in Fig. 1. We first measured cutaneous sensitivity on the dominant limb plantar foot surface at the first metatarsophalangeal joint and central aspect of the heel using the Semmes–Weinstein monofilament test (Nakamoto et al. 2022). To account for interrater unreliability and subjectivity of detection thresholds, a 4-2-1 stepping algorithm approach was followed in the application of monofilament pressure tests using a standard Semmes–Weinstein test kit (Texas Medical Design, Inc., Sugar Land and Stafford, Texas) (Snyder et al. 2016).

 We then recorded preferred walking speed for all participants as the mean of four passes down a 30-m hallway using photocell timing gates (Bower Timing Systems, Draper, Utah, USA). We collected surface electromyography (EMG) using Trigno Avanti electrodes (10 mm inter-electrode distance; Delsys Inc., MA, USA) from the LG, SOL, and TA muscles of the dominant leg during walking using in accordance with SENIAM guidelines (Hermens et al. 2000). Before placing electrodes, the skin was shaved to remove hair and cleansed with isopropyl alcohol wipes. For the LG, electrodes were placed in parallel with fiber orientation over the most prominent bulge of the muscle belly as identified through palpation during standing plantarflexion. For the SOL, electrodes were placed in parallel with fiber orientation over the muscle belly on the lower lateral third of the line connecting the medial femoral condyle to the medial malleolus, also palpated during standing plantarflexion. For the TA, electrodes were placed in parallel with fiber orientation over the muscle belly on the upper third of straight line connecting the tibial tuberosity to the medial malleolus, palpated during standing dorsiflexion. Participants then completed a 3-min warm-up walking trial on a dual-belt, instrumented treadmill at their preferred walking speed (Bertec, Columbus, Ohio, USA). This was followed by a single walking trial where participants walked at their preferred speed while responding to a series of five unanticipated treadmill-induced slip perturbations. For each, the treadmill belt under the right foot (dominant foot in all participants) decelerated at a rate of 6 m/s^2^ for 200 ms (based on preferred walking speed, mean displacement – OA: 12.4 cm, YA: 14.7 cm), after which participants returned to their preferred walking speed (PWS) after at least 10 steps to ensure gait had normalized prior to the next perturbation (Crenshaw and Grabiner 2014). While all perturbations were triggered at heel strike, there was a ~ 40 ms delay from heel strike until the belt deceleration began. Heel strike and toe-off events were determined using a 20 N threshold on the vertical ground reaction forces, conditioned using a zero-lag Butterworth filter with a low-pass cut-off of 50 Hz.

Finally, during two passive, isokinetic ankle rotations (20°/s from 20° plantarflexion to 30° dorsiflexion), we recorded the distal medial gastrocnemius musculotendinous junction (MTJ) with the Achilles tendon (AT) using B-mode ultrasound images. All passive rotations were performed on a dynamometer (Biodex System 4 Pro, Shirley, New York, USA) with the knee flexed to 20°. All ultrasound images were collected via a 60 mm linear array transducer (LV7.5/60/128Z-2, UAB Telemed, Vilnius, Lithuania) operating at 61 fps and imaging at a 50-mm depth adhered to the posterior aspect of the shank with a custom 3D-printed holder and self-adhesive bandaging. Simultaneously and in synchrony, using a 15-camera motion capture system (Motion Analysis Corporation, Rohnert Park, CA, USA), we collected 3d positions of retroreflective markers on each participant’s calcaneus and a 3-marker cluster fixed to the ultrasound probe.

### Cutaneous sensitivity and Achilles tendon stiffness

Semmes–Weinstein monofilament heel and toe thresholds were combined into a single average value per participant. Given the relevance of sensing initial unanticipated changes in ankle joint posture, we calculated passive k_AT_ according to previously described methods (Smith et al. 2024) from the average of the two passive ankle dorsiflexion rotations. Here, the intersection of the gastrocnemius and AT (i.e. MTJ) was manually labeled and tracked (Tracker, V 6.0.10). We labeled every fifth point in sequential b-mode images and interpolated MTJ position throughout the entire rotation due to the relatively linear trajectory of the MTJ during isokinetic rotations (Krupenevich et al. 2020). The MTJ position data were then transformed into a common coordinate system with the ultrasound probe, and the 3D distance between the MTJ and calcaneus position was calculated as AT length. AT force was then calculated as the dot product of dynamometer torque and AT moment arm length, for which we used a constant value from literature (Rasske and Franz 2018). We then calculated k_AT_ as the slope of linear best fit of the dividend of AT force and AT length from 20 to 80% of passive ankle dorsiflexion range to isolate the linear region of the tendon’s stress/strain curve.

### Muscle excitation responses

In order to maximize signal-to-noise ratios and improve detection of muscle onset times, EMG data for all muscles (i.e. TA, SOL, MG) following the treadmill-induced slip perturbations were processed according to previous methods (Solnik et al. 2010). Each signal was high-pass filtered at 20 Hz to remove motion artifacts, then transformed by a Teager-Kaiser energy (TKE) operator. The TKE operator provides a signal proportional to the instantaneous amplitude and frequency of the input EMG, effectively amplifying the motor unit voltage spikes (Solnik et al. 2010). Therefore, we calculated muscle excitation onset times for each muscle’s waveforms using the following criteria: (1) onsets must occur beyond 50 ms following perturbation application, eliminating early onset detection from pre-activation at heel strike; (2) onsets appearing reflexive (i.e. EMG burst earlier than 50 ms following perturbation application) must exceed 3 standard deviations above signal mode for more than 25 frames (Solnik et al. 2010); and (3) onsets must follow ankle joint rotation thresholds ≥ 3° as evidence of MTU length change. Of note, criteria 2 differs slightly from that of Solnik et al. (Solnik et al. 2010), as they considered 3 standard deviations from the signal mean 400–500 ms prior to a known onset of their simulated signal (i.e. analogous to perturbation onset here). However, as our EMG signals were collected during walking, they were relatively noisier and without a consistent quiet phase before perturbation onset. Instead, we opted to use the mode of the EMG signal from the entire perturbed walking trial per muscle, allowing us to establish a consistent reference signal while accounting for well-established increases in baseline EMG signal noise under perturbed conditions as compared to habitual (Marigold and Patla 2002). Excitation onset times for all muscles are reported as the time from perturbation application (~ 40 ms following heelstrike) until the onset conditions were met. EMG onset times for each muscle are reported as group averages for older and younger adults, with each group average representing the mean of individual values across five perturbation trials.

### Statistics

Independent samples t-tests were performed to determine the effects of age on muscle onset times, PS, and k_AT_ (SPSS V28, Chicago, Illinois, USA). Shapiro-Wilkes tests for normality were performed for all variables (*p* > 0.05). For all t-tests, we included PWS as a covariate due to age-related differences in habitual walking speed and their potential effect on muscle onset timing. Then, we performed multivariate linear regressions to assess whether PS and k_AT_ were associated with muscle excitation responses across our entire cohort and within groups. All statistical significance was set at *p* ≤ 0.05, and group results are reported as mean values ± standard deviations with effect sizes (i.e. *d*).

## Results

### Between-group differences

**Fig. 2:**
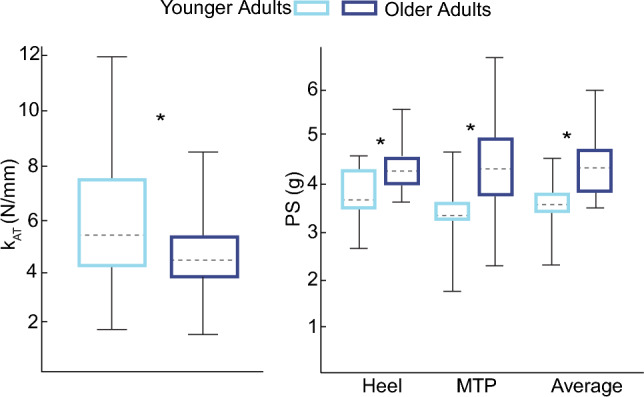
Achilles tendon stiffness and Semmes–Weinstein thresholds. Group average data for younger (light blue) and older (dark blue) adults for Achilles tendon stiffness (k_AT_) and plantar sensation (i.e., Semmes–Weinstein) thresholds (PS). Significant between-group differences are denoted by single asterisks (*), defined using a critical alpha value of 0.05

All variables were normally distributed. OA had slower PWS than YA (OA:1.22 ± 0.16 m/s, YA: 1.34 ± 0.12 m/s). OA had higher plantar Semmes–Weinstein thresholds than YA at the heel (OA:4.37 ± 0.46 g, YA:3.77 ± 0.50 g, *p* < 0.001, *d* = 1.26) and first metatarsophalangeal head (OA = 4.47 ± 0.99 gm, YA = 3.22 ± 0.60 gm, *p* < 0.001, *d* = 1.55) (Fig. 2). The average of these heel and toe values, which we used in subsequent correlations, also significantly differed between groups (OA: 4.42 ± 0.67 g, YA: 3.50 ± 0.50 g, p < 0.001, d = 1.53). OA also had lesser passive k_AT_ than YA (OA: 4.44 ± 1.87 N/mm, YA: 6.12 ± 2.82 N/mm, p = 0.033, d = 0.70) (Fig. 2).

**Fig. 3:**
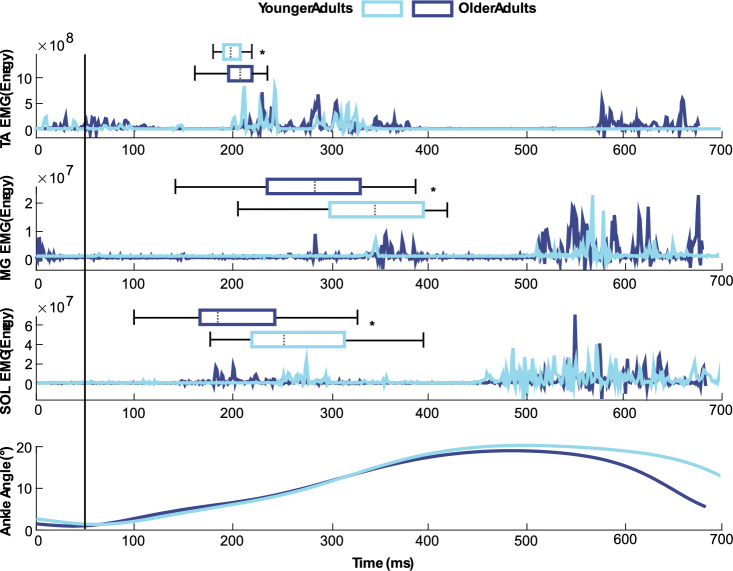
Muscle and ankle joint angular responses to treadmill-induced slip perturbations. Time series data from heel strike until toe-off of a perturbed step for a representative older (dark blue) and younger (light blue) adult for medial gastrocnemius (MG), soleus (SOL), and tibialis anterior (TA) muscle excitations and group mean ankle angles. Group mean muscle onset times are shown as horizontal box-and-whisker plots. Perturbation onset is shown as a solid, vertical black line. Significant between-group differences are denoted by single asterisks (*), defined using a critical alpha value of 0.05

TA onset times following slip perturbations were slower for older than YA (OA: 163.93 ± 21.71 ms, YA: 151.21 ± 9.31 ms, *p* = 0.008, *d* = 0.76) (Fig. 3). Conversely, albeit later in the gait cycle than for the TA, OA had earlier onset times for the MG (OA: 234.99 ± 77.35 ms, YA: 310.25 ± 64.61 ms, *p* < 0.001, *d* = 1.06) and SOL (OA: 161.01 ± 71.17 ms, YA: 204.13 ± 67.54 ms, *p* = 0.027, *d* = 0.62) muscles (Fig. 3). As treadmill induced perturbations were administered at participants’ preferred walking speeds, and OA walked slower than YA (*p* = 0.05, *d* = 0.83), we report the same group comparisons with walking speed as a covariate, where OA had delayed TA excitation onsets (*p* = 0.032, η_p_^2^ = 0.17), earlier MG excitation onsets (*p* = 0.07, η_p_^2^ = 0.23), and no difference in SOL excitation onsets (*p* = 0.175, η_p_^2^ = 0.09) compared to YA when accounting for walking speed.

**Fig. 4:**
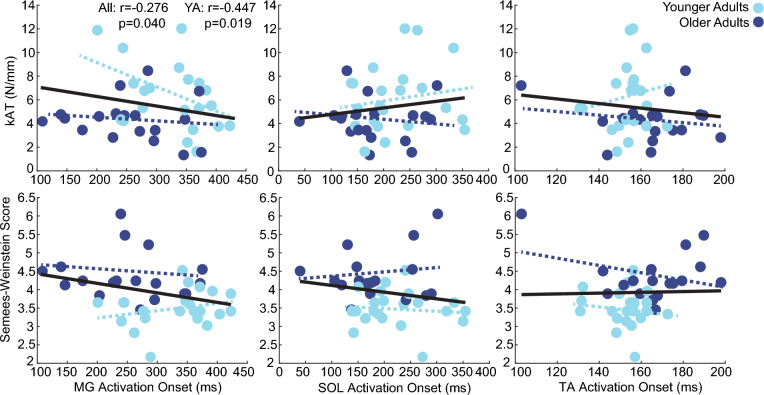
Series elastic stiffness and plantar sensitivity relations to muscle onset times. Scatter plots of the individual associations between muscle excitation and Achilles tendon stiffness (k_AT_, top row) and plantar sensation (i.e., Semmes–Weinstein) thresholds (PS, bottom row) for the medial gastrocnemius (MG), soleus (SOL), and tibialis anterior (TA) for younger (light blue) and older (dark blue) adults. Significant associations are denoted by single asterisks (*), defined using a critical alpha value of 0.05

Across our cohort and in OA, we found no significant correlations between monofilament thresholds or k_AT_ and onset time for any instrumented muscle (Table 1). The same was true for SOL and TA onset times in YA. Conversely, we found a significant association for MG in YA (F(2,19) = 3.759, *p* = 0.042, *R*^*2*^ = 0.284), where earlier excitation was associated higher k_AT_ (*r* = − 0.447, *p* = 0.019) (Fig. 4) but not lower monofilament thresholds (*r* = 0.246, *p* = 0.135). The distributions of all individual relations are shown in Fig. 4.

**Table 1:** Linear regression model summaries and individual correlation values for muscle excitation onset times for the medial gastrocnemius (MG), soleus (SOL), tibialis anterior (TA) and plantar sensitivity (PS) and Achilles tendon stiffness (k_AT_)

		All			OA			YA		
		PS	k_AT_	Model	PS	k_AT_	Model	PS	k_AT_	Model
MG	*r*	− 0.276	− 0.085	0.288	− 0.117	− 0.105	0.124	0.246	− **0.447**	**0.532**
	*p*	0.080	0.298	0.193	0.316	0.334	0.883	0.135	**0.019**	**0.042**
	F			1.719			0.125			3.759
SOL	*r*	− 0.160	0.170	0.235	0.142	− 0.044	0.222	− 0.093	0.154	0.188
	*p*	0.319	0.145	0.341	0.281	0.429	0.667	0.341	0.246	0.709
	F			1.106			0.415			0.350
TA	*r*	0.025	− 0.131	0.134	− 0.306	− 0.192	0.306	− 0.113	0.164	0.208
	*p*	0.877	0.207	0.709	0.101	0.216	0.455	0.309	0.233	0.656
	F			0.348			0.827			0.431

## Discussion

We sought to determine the roles of series elastic tissue stiffness and plantar cutaneous sensitivity on muscle response times during initial recovery from treadmill-induced slip perturbations in OA and YA. Consistent with our hypotheses, we demonstrated hallmark age-related decreases in PS and k_AT_ and revealed delayed TA muscle onsets in OA in response to treadmill-induced slip perturbations. Given the nature of ankle kinematic changes following slip perturbations agnostic to age, the burst of TA excitation following perturbation onset may stem from delayed dorsiflexion compared to what would be expected during an unperturbed stride. With no clear evidence of rapid TA muscle stretch regardless of age, we suggest these early signals originate from cutaneous stimuli, Golgi tendon organ feedback, or non-reflexive muscle spindle feedback. Later in the stance phase and contrary to our hypotheses, OA had earlier SOL and MG excitation onset times than YA following perturbations. As we discuss later, these differences may reflect a proclivity for increased antagonist coactivation in OA and/or more effective reciprocal Ia inhibition in YA. We were unable to establish a definitive link between muscle reflex delays and age-related decreases in Achilles tendon stiffness and plantar sensitivity. However, we did find that YA with greater k_AT_ had faster MG onsets, which warrants future investigation for its potential to explain subsequent balance recovery strategy and efficacy. Ultimately, our results point to specific age-related changes in the timing of neuromuscular corrections to mitigate instability, which may underlie age-related differences in balance recovery efforts and subsequent injury severity.

In this study, we employed treadmill belt decelerations (i.e. slips) to perturb balance, allowing measurement of muscle responses indicative of perturbation detection. Similar to previous work by Tang et al., we found delayed TA excitation onsets (11 ms in Tang et al., 12 ms here) following slip perturbations in OA versus YA (Tang and Woollacott 1999). Considering differing slip paradigms and perturbation magnitudes between this study and that of Tang et al., our combined results indicate a robust reflex delay relevant to detecting slip-like balance perturbations due to age, even when accounting for age-related differences in preferred walking speed (Tang and Woollacott 1999). Notably, while we originally anticipated the perturbation to hasten forefoot contact (i.e. increase ankle plantarflexion velocity) following heelstrike to trigger a TA stretch reflex, our data showed that perturbation onset occurred immediately before or during forefoot contact for all participants. Accordingly, these ankle joint profiles fail to show evidence of rapid TA elongation necessary to implicate the TA stretch reflex in either age group. Instead, the perturbation acted to delay ankle dorsiflexion compared to what would be expected during an unperturbed stride (see Supplemental Figure). While joint capsule receptors signal primarily only at extreme positions or velocities (Burke et al. 1988), we posit that the TA excitation induced by our perturbations arises from more subtle muscle length or tension changes (i.e. Golgi tendon organ reception) or sensation from plantar cutaneous receptors.

This study and others report relatively long-latency (i.e., > 100 ms) TA excitation during quiet stance (Obata et al. 2012), habitual walking (Christensen et al. 2001), and following slip perturbations (Tang and Woollacott 1999). This muscle onset timing implicates neural integration between muscle stretch reflexes and cortical pathway involvement, whose combined reflex responses are known to be vital during stabilization efforts of the support limb during stance in walking (Christensen et al. 2001). Thus, age-related delays in TA excitation onset stemming from muscle spindle feedback likely originate from a combination of reduced muscle spindle acuity and slower cortical processing/sensory integration with aging. Nevertheless, given OA’ predisposition for falls and fall-related injuries, delays in TA excitation onset may obstruct detection of a balance perturbation and should be examined for their potential role in worsening subsequent balance recovery efforts among OA.

Past work investigating reflex responses in aging have reported delayed onset of reflex responses for the MG and SOL for perturbations that elicit the opposite change in joint position as our paradigm – namely, trips and obstacle crossings (Pijnappels et al. 2005). Based on an assumption of generalized delays due to age, we hypothesized that SOL and MG excitation onsets in OA would also be delayed compared to YA. Instead, we found that OA exhibited earlier MG and SOL excitation onsets following slip perturbations. Also, these muscle responses were temporally removed from initial dorsiflexion and thus were unattributable to reflex activity per se. In light of delayed TA excitation, which acts as an agonist to the slip perturbation, earlier MG and SOL activity among OA may be a compensatory mechanism during early balance recovery, as subsequent propulsion is a vital component of repositioning the center of mass to arrest instability (Vlutters et al. 2016). OA are also known to coactivate their distal leg muscles more than YA during habitual walking (Schmitz et al. 2009) and when walking while responding to balance perturbations (Acuña et al. 2019). Antagonist coactivation is often interpreted as a neuromuscular strategy to increase joint impedance and thus stability (Nagai et al. 2013), where earlier SOL and MG excitation onsets for OA could act to counteract the instability elicited by slip perturbations. Though, our data (see Supplemental Table) show OA activate their SOL and MG muscles earlier during unperturbed walking as well, implying that this strategy is not unique to perturbation responses. Two other plausible interpretations exist. The first is that age-related reductions in rate of force development (Izquierdo et al. 1999) may require earlier triceps surae excitation in OA to propel walking and recover stepping, independent of perturbations. The second is that YA may have more effective reciprocal Ia inhibition than OA. Indeed, particularly given the agonist nature of the TA during the early perturbation response, simultaneous inhibition of the MG and SOL would also explain later onsets for these muscles in YA.

OA displayed lesser k_AT_ than YA, a common finding in literature (Delabastita et al. 2019). However, interindividual differences in k_AT_ were not associated with muscle excitation onsets following perturbations. One explanation is that our perturbation may not have provided sufficiently rapid stimuli via TA elongations to elicit a stretch reflex response our premise contends would be mediated via series elastic stiffness. Nonetheless, these results echo those of Blackburn et al., who found no association between active triceps surae MTU stiffness and SOL short-latency reflex responses (~ 50 ms) during rapid, dorsiflexion perturbations in a dynamometer (Blackburn et al. 2008). Therefore, we contend that age-related delays in reflex excitation in ankle musculature reported elsewhere (Obata et al. 2012) and in our data are more likely explained by concurrent age-related decreases in intrafusal fiber number and integrity (Liu et al. 2005) than by age-related decreases in Achilles tendon stiffness. In our findings and those of Blackburn et al. (Blackburn et al. 2008), tendon slack length was taken up either by pre-tension in the TA during walking or in the SOL during discrete ankle plantarflexion dynamometer rotations, respectively. Unlike the conditions of our study and that of Blackburn et al. the triceps surae of the stance limb are relatively inactive (i.e., no Achilles’ tension) when the contralateral foot is exposed to a trip perturbation (Pijnappels et al. 2005), which may represent an epoch where series elastic stiffness is indeed able to impact muscle stretch magnitude and velocity. Thus, it is still conceivable that reflex delays could occur due to decreased tendon stiffness under conditions where the tendon slack length is unattenuated during the gait cycle.

We did find that, in partial support of our hypothesis, YA with greater k_AT_ had faster MG onsets following treadmill-induced slip perturbations. Again, considering the relative timing of SOL and MG onsets in relation to perturbation-induced changes in ankle kinematics, we are not confident interpreting greater k_AT_ as facilitating faster stretch reflex activity from the triceps surae muscles. Instead, presuming a stiffer tendon enables faster force transmission and perhaps therefore more effective net propulsive impulse during late stance, YA with greater k_AT_ may exhibit earlier SOL/MG excitation to increase forward propulsion. We would interpret this as evidence that YA with stiffer tendons may rely more on an ankle-dominant strategy deployed to recover from the instability elicited by slip perturbations (Hwang et al. 2009).

Consistent with prior reports, OA in our study required thicker Semmes–Weinstein monofilaments to detect plantar stimuli than YA (Perry 2006; Peters et al. 2016). However, this decline in sensory acuity was not related to delays in TA excitation following treadmill-induced slip perturbations. Past studies using a range of vibratory thresholds have demonstrated decreased PS with age (Perry 2006), particularly for vibrational frequencies near 30 Hz, which stimulate the same fast adapting type-I cutaneous mechanoreceptors that are targeted by monofilament testing. However, higher vibration frequencies (100–250 Hz) stimulate fast adapting type-II afferent receptors, which are more closely correlated with balance control among older adults (Peters et al. 2016). It seems even more likely that type-II afferent receptors would be stimulated during an early balance recovery. Thus, while monofilament testing is clearly sensitive to detection of sensory acuity declines with age, the afferent receptors stimulated by monofilaments may not directly govern ankle muscle reflex thresholds during balance recovery. Of note, given the concentration of fast adapting type-II afferent in the foot and heel regions (Strzalkowski et al. 2017) and their potential relevance to balance recovery efforts, future research may gain insight into the precise determinants of balance recovery strategies by investigating the relationship between muscle reflexes and higher frequency stimulations at the heel and toe regions.

There is an experimental limitation to consider when interpreting our results. Our K_AT_ values are relatively small in comparison with previous literature (Delabastita et al. 2019). To isolate the tendon’s mechanical contribution to reflex responses following slip perturbations, we measured kAT during passive dorsiflexion rather than active conditions. This approach allowed us to assess the tendon’s inherent stiffness independent of the confounding influence of active muscle stiffness, which would better replicate a reflexive response to an unanticipated perturbation at heel strike. As such, due to the relatively low k_AT_ values in our data, we likely included the toe region of tendon elongation in our calculations. Also, to address general age-related differences in muscle onset times following gait slip perturbations, we chose to analyze the average of five perturbation trials. However, we acknowledge previous literature has shown muscle responses can change following repeated exposure to gait perturbations, which our approach does not consider.

In conclusion, we have shown that age-related declines in plantar sensory acuity and musculotendinous integrity accompany age-related delays in TA reflex responsiveness following treadmill-induced slip perturbations. While neither subcutaneous sensitivity nor series elastic tissue stiffness were statistically associated with delayed muscle reflex activity, we encourage future studies that include higher-frequency cutaneous stimulations and the explore the effects of age-related delays in muscle reflexes on subsequent balance recovery efforts and falls risk in older adults.

## Supplementary Information

Below is the link to the electronic supplementary material.

Supplementary file1 (DOCX 85 KB)

## Data Availability

A master data file is available here: https://dataverse.unc.edu/dataset.xhtml?persistentId=doi%3A10.15139%2FS3%2FCWFKIN&version=DRAFT.
