# Supplementary material for: The effects of series elastic stiffness and cutaneous sensitivity on leg muscle reflex responses to unanticipated slips during walking
Source: Exp Brain Res. 2025 May 20;243(6):150. doi: 10.1007/s00221-025-07095-8 (PMC12092490; doi:10.1007/s00221-025-07095-8)
Supplement: Supplementary file 1 — Supplementary file1 (DOCX 85 KB) [file 221_2025_7095_MOESM1_ESM.docx]

**Supplement for “The effects of series elastic stiffness and cutaneous sensitivity on leg muscle reflex responses to unanticipated slips during walking”**

**Supplemental Table.** Groups means, standard deviations, and independent t-test results for soleus (SOL) and medial gastrocnemius (MG) muscle excitation onset times during unperturbed walking for younger and older adults. Significant results are denoted with bold text with a critical alpha level of 0.05.

|  | OA | |  | | YA |  |
| --- | --- | --- | --- | --- | --- | --- |
| Unperturbed EMG Onsets | | Mean ± SD | | | | *p* |
| SOL Onset Time (ms) | **124.68 ±** **45.97** | | | **167.7 ± 69.92** | | **0.028** |
| MG Onset Time (ms) | **177.34 ± 73.85** | | | **220.7 ± 49.83** | | **0.031** |

**
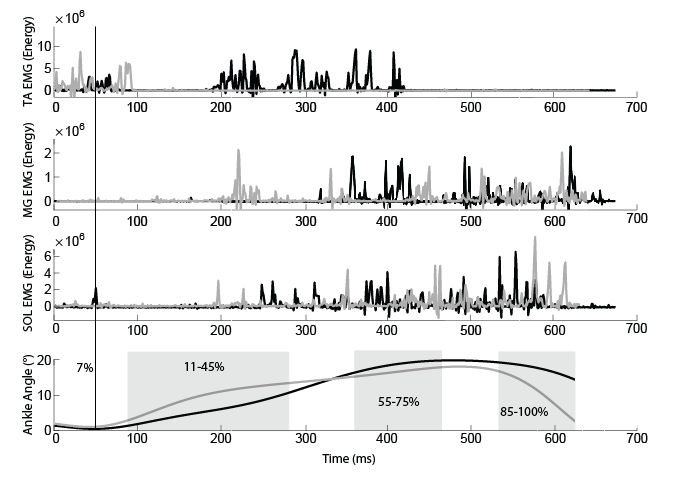
**

**Supplemental Figure. Ankle muscle excitation and ankle angles during habitual and perturbed gait.** Representative time-series data over stance from a younger adult from heel strike until toe-off for habitual (grey) and perturbed (black) stance for the medial gastrocnemius (MG), soleus (SOL), and tibialis anterior (TA) muscles. The bottom panel depicts mean ankle angles for habitual and perturbed gait with older and younger adults’ data combined per walking condition over adjusted stance. Stance was adjusted by removing 48 frames of perturbed stance to ensure equal time periods, allowing statistical parametric mapping comparisons between continuous time-series data. Shaded regions show regions as percent of stance where ankle angle trajectories significantly differ between walking conditions. Perturbation onset timing for the perturbed step is shown as a vertical black line. (p=0.05)

**Supplemental Methods**

*Ankle Kinematics*

We captured 3D positions of retroreflective markers placed on the shank, heel, and feet to define foot and shank segments for calculation of ankle kinematics during walking. Marker trajectories were exported to Opensim (v4.1), scaled according to individual anthropometry), and inverse kinematics for the ankle were calculated using a generic musculoskeletal model (Gait2392. Scaling and inverse kinematics were conducted using Opensim defaults, Scale and Inverse Kinematic Tools. All marker data was filtered at 6 Hz using a zero-lag 4^th^ order Butterworth filter and joint angles were extracted.

*Statistics*

Two-tailed statistical parametric mapping (SPM) independent sample t-tests were performed in Matlab using the spm1d (Patacky, 2010) (version M.0.4.7, www.spm1d.org) to assess the effect of treadmill-induced slip perturbations on ankle angle of the perturbed leg. Younger and older adults data over the 5 discrete perturbations were averaged per participant and combined per condition (habital and perturbed). As SPM requires data being compared to be of equal length and were were interested primarily in the initial effects of the perturbation on ankle kinematics, we removed the final 48 frames of the ankle angle for perturbed stance (i.e. adjusted stance) to equalize ankle angle vector lengths between habitual and perturbed conditions.

Two tailed independent sample t-tests were performed to determine the effect of age on muscle onset times for the MG and SOL during habitual walking. (SPSS V28, Chicago, Illinois, USA). Shapiro-Wilkes tests for normality were performed for all variables. We included walking speed as a covariate due to age-related differences in habitual walking speed and their potential effect on muscle onset timing. Alpha for statistical significance for *p*-values were set at ≤ 0.05 for all analyses.

*Supplemental Results*

All variables were normally distributed per Shapiro-Wilkes’ test of normality. Ankle angles following the perturbation was significantly less dorsiflexed from 11-45% of adjusted stance (*p*<0.001) and significantly more dorsiflexed from 55-75% (*p*=0.006) and 85-100% (*p*=0.015) of adjusted stance when compared to ankle angles during habitual walking (t*=2.951) (Supplemental Figure).

MG and SOL onset times during habitual walking were significantly earlier in older adults when compared to younger adults (MG: *p* = 0.031, d = 0.73, SOL: *p* = 0.028, d = 0.69) (Supplemental Table).
